# Reflex Paraplegia—Reprinted from *Braithwaite*

**Published:** 1862-03

**Authors:** 


### REFLEX PARAPLEGIA.

BY THE EDITOR OF THE BRITISH MEDICAL JOURNAL.

Reprinted from *Braithwaite*.

Perhaps one of the earliest and ablest writers on reflex paraplegia is Dr. Graves, in his lectures on paraplegia (1843).

Dr. Brown-Séquard is, however, the latest writer on the subject. He gives us an excellent account of the affection, and also a novel explanation of its nature—an explanation, however, which we fear must, for the present, remain hypothetical. Let us see what it is he tells us on this important subject.

The two most frequently observed and clearly defined forms of paraplegia are, reflex paraplegia, and paralysis depending upon myelitis or disease of the spinal cord. Heretofore there has been no proper line of distinction drawn by pathologists between these two varieties of paralysis; but this distinction is absolutely necessary for the important purposes of rightly directing the practitioner's treatment; medicines which are suitable in the one kind being injurious in the other.

Paraplegia depending upon disease or injury of the spinal cord has been long well understood by us all; but what is meant by *reflex paraplegia?* Is there really such a disease? By reflex paraplegia is meant that kind of paralysis of the lower limbs which results from an excitation of the spinal cord, the excitation being conveyed to the spine through a sensory nerve. For example, some part of the body, say the uterus, is irritated, injured, or displaced; the effects of the displacement, etc., are appreciated by sensory nerves, and are thereby conveyed to the spinal nervous centre, and, by the action which they there produce, cause those changes to occur which result in paraplegia. According to Dr. Brown-Séquard, the paralysis is produced in the following way. The excitation aforesaid acts either upon the blood-vessels of the spinal cord itself or upon those of the motor nerves proceeding from it, or upon those of the muscle. This is what, pathologically speaking, is meant by reflex paraplegia. Through this excitation, spasmodic contraction of the blood-vessels is produced, and consequently a deficient supply of blood is sent to one or other of the aforesaid parts.

Dr. Brown-Séquard has, however, first of all, before proceeding to speak of its treatment, etc., to answer the objections of those who deny the existence of the affection; and there are a few men of high repute who do deny the thing. The answer of Dr. Brown-Séquard is, on the face of it, the best which can be given. He leaves transcendental physiology, and asks his readers to look at a plain series of facts which he unrolls before their eyes, and which are comprehensible to the understanding of the youngest and the oldest of us. To us, we must confess, these facts demonstrative are as "plenty as blackberries;" and we cannot imagine that any one except a *tête monté* physiologist, or an absolute specialist can refuse to accept the consequences which flow from them. A female suffers from paraplegia. Her uterus is displaced. The abnormal position of it is rectified, and the paralysis disappears. Amaurosis exists in combination with irritation of the frontal nerve; the frontal nerve is divided, reflex action from the irritated spot is cut off, and sight rapidly returns. Neuralgia of external parts exists: and the patient's auditory powers are good, bad, and indifferent, according to the absence, presence, and acuity of the neuralgia. Dentition, worms, irritation of the womb, diptheritis, etc., are at times associated with various kinds of paralysis; and *sublatâ causâ tollitur effectus* has been duly observed in such cases.

Now, the proof that these paralyses do not depend upon disease of the nervous centres is this very plain one; viz., that the removal of the peripheric irritation—the worms, the displaced womb, etc.—could not in such case have removed the paralysis. And if a very hardened sceptic in these matters should still object that, nevertheless, the cures in such cases might have been more properly called rather a coincidence than a consequence, we should advise him to consult Dr. Brown-Séquard's book, and study his witnesses. The collection of cases there to be found, derived from numerous different sources, will, we fancy, demonstrate to the satisfaction of every one—except those who, apparently for argument's sake, will on occasion deny that B follows A in the alphabet—that *reflex paralysis* is a medical fact.

In these cases it will be seen, that what Dr. Brown-Séquard calls the *outside irritation* preceded the paraplegia, and that cure of the latter quickly followed the removal of the irritation. Again, in many of these cases, the paralysis increases and decreases with the condition of acuity of the outside irritation; and no treatment has any effect in removing the paralysis so long as the irritation was left unremoved. In some of the cases the paraplegia appeared, disappeared, and reappeared several times over with the appearance, disappearance, and reappearance of the outside irritation. "Can there," says Dr. Brown-Séquard, "be more decisive proofs than that it is the outside irritation starting from some sensitive nerves in various parts of the body, which produces the paraplegia?"

Besides, if we do not accept this view of the case, we are left without any means of explanation of the paralysis in most of these cases. The idea of these kinds of paralysis being produced by, for instance, pressure of the uterus on the obturator nerves or on the sacral plexus; or by the action of poisonous matters accumulating in the blood in retention of urine, or in diptheritis, or enteritis, is very chimerical.

But, it is objected, we cannot accept this reflex paraplegia; for it is impossible to understand how it can occur. And here the physiologist steps in, and answers without hesitation—*les pieces á la main*—that on the contrary, it is very easy to show how a paralysis can take place by a reflex action starting from a sensitive nerve. And practitioners who pride themselves on being practical men, and *not* physiological physicians, must, we suppose, here take a lesson from the physiologist.

An outside irritation may produce paraplegia by *causing reflex contraction of blood-vessels* in two ways. It is proved beyond doubt, that blood-vessels have similar relations to the nervous system, to those which muscles of animal life have to this system. Blood-vessels contract with energy and sometimes pass into a state of prolonged spasm: 1. When their motor nerves are *directly* excited; and 2. When excited through the cerebro-spinal axis through irritation applied to some centripetal or excito-motor nerve. In *these contractions of blood-*

*vessels* we find the cause of paralysis. The contractions may occur: 1. In the blood-vessels of the spinal cord; 2. In the vessels of the motor nerve passing from the cord; and 3. In the vessels of the muscles themselves.

Dr. Brown-Séquard has, he tells us, seen contraction of the blood-vessels of the pia mater took place in the spinal cord, when the lobes of the kidney, or the vessels and nerves of the suprarenal capsules, were ligatured; and the contraction is generally greatest on that side of the cord corresponding with the side irritated. Paralysis of the lower limb has been known to follow extirpation of the kidney on the same side of the body. Again, reflex paraplegia may be produced by *insufficiency of nutrition*, resulting from this contraction of the blood-vessels. This explanation of the paraplegia as being a consequence of contraction of blood-vessels, must however, at present be received as theoretical.

Another argument in favor of this view of the case is derived from the fact of the normal condition of the spinal cord —the absence of all visible signs of disease—observed in persons who have died after presenting symptoms of reflex paraplegia associated with retention of urine.

Thus, then, here stand the facts touching Reflex Paraplegia: 1. There is no organic disease of spinal cord; 2. The paralysis will rapidly disappear when the source of reflex irritation is cut off; and 3. The vascular supply of cord, of motor nerves, and of muscles is under the influence of reflex action.

And now then, we see the grand anatomical distinction between reflex paralysis, and paralysis associated with myelitis. In the first case, there is diminished, in the second, increased vascularity of the cord. In myelitis, the color, the consistency, and the vascularity of the cord, are visibly changed. In reflex paraplegia, these qualities of the cord are found, to all appearance, unaltered after death. And here, therefore, as Dr. Brown-Séquard tells us, we have at once before us two groups of paraplegic diseases—one in which too much blood is circulating in the spinal cord, and one in which the very opposite condition of the cord exists. And then, he adds, consider the value of a right discrimination between them. The remedies which suit one group are actually injurious to the other. There are some, and he says that he can prove the fact, that diminish the amount of blood in the spinal cord; and there are others that increase the quantity. And yet, these remedies are often blindly and indiscriminately employed in paraplegia. Mercury, ergot of rye, and belladonna, Dr. Brown-Séquard asserts diminish the blood, and strychnine and brucia increase the blood in the spinal cord.

[In most cases, reflex paraplegia may be characterized as follows:—

There is an absence of the special symptoms which indicate organic disease of the spine; the paralysis is incomplete, and has slowly supervened upon an affection of an urinary, a genital, or some abdominal organ, upon inflammation of the lungs or pleura, or after irritation of a nerve in its trunk or cutaneous ramifications. In most cases of paraplegia, we must form our judgment as to its nature from a consideration of these conditions.

The following are the distinctions which Dr. Brown-Séquard has collected together and given as characteristic of the two best marked varieties of reflex and centric paraplegia—urinary paraplegia and myelitic paraplegia.

*Urinary paraplegia* is *preceded* by an affection of the urinary organs. The paralysis is incomplete, and varies in degree with the varying conditions of these organs. The lower limbs alone are usually affected, and the paralysis does not extend upwards. Reflex power is not wholly lost or is much exalted. Rarely do spasms seize the paralyzed muscles; nor is there pain in the spine, nor sense of constriction of the abdomen; neither formication, nor pricking, nor anæsthesia. Rarely is the bladder or rectum paralyzed; there is much gastric disturbance; and lastly, recovery often takes place rapidly, after notable amelioration of the urinary affection.

On the other hand, in *myelitic paraplegia*, the affection of the urinary organs does not *precede*, but is a *consequence* of, the spinal disease. The paralysis is complete, and is unaffected by changes in the condition of the urinary organs. Other parts beside the lower limbs are usually paralyzed, and paralysis gradually extends upwards. Reflex power is often lost, but sometimes much increased. There are always spasms and some amount of pain; also formication, pricking, etc.; feeling of abdominal constriction, and almost always anæsthesia or numbness. There is no gastric disturbance. The disease usually slowly progresses to a fatal termination, and rarely ever is there a complete recovery. Moreover, the urine in myelitis is alkaline; whilst in reflex paraplegia not depending upon urinary diseases it is usually natural.

In considering the *prognosis* of reflex paraplegia, we must, according to Dr. Brown-Séquard, reflect that there are two forms of the affection; that in one of these forms, the paraplegia depends upon a defect in the nutrition of the spinal cord; and in the other upon an alteration of nutrition in the muscles of the lower limbs. The first of these forms is almost always curable, provided the external irritating cause be removable. But, in the second case, recovery rarely takes place, even though the provoking cause of the paraplegia be removed; and the reason is, that in this form the muscles have become rapidly atrophied and altered. Hence as a matter of prognosis, it is always important to know, in a case of reflex paraplegia, whether or not rapid atrophy of the muscles has occurred.

The *treatment*—or to speak more correctly, the indications for treatment, of reflex paraplegia spring naturally from the pathology of the disease as here laid down. The first indication evidently is to remove the local irritation, the cause of the affection; and this necessarily involves the consideration of the treatment of nephritis, cystitis, enteritis, morbid states of the uterus, in fact, of all those morbid conditions with which the paraplegia may be associated. In carrying out this treatment, we must also endeavor to diminish the sensibility, or interrupt the nervous influence which passes from the diseased organ or nerve to the spinal cord, to paralyze the nervous sensation. For this purpose says Dr. Brown-Séquard, no narcotising agent is more powerful than belladonna; but, in some cases, caution is required in its use. In disease of the uretha, or prostate, one grain of the extract of belladonna in twenty drops of laudanum should be injected into the urethra, and washed away after half-an-hour or an hour's residence there. This treatment should be repeated every two or three days; thirty drops of laudanum alone being injected on the intervening days. In bladder-disease, the aforesaid solution of belladonna is to be injected after evacuation of the urine; and then the sole laudanum injection on alternate days. Suppositories of these two agents may also be employed.

If the irritation radiate from the vagina or uterus, then a pill of half a grain of belladonna, with one grain of extract of opium, enclosed in a piece of cotton wool, may be passed well up into the vagina—a practice of M. Trosseau's, which Dr. Brown-Séquard much extols. Should the stomach, or the intestines, or kidneys, etc., be the seat of local irritation, then opium should be taken by the mouth.

Dr. Graves has strongly recommended excitants or revulsives to be applied to the skin of the legs; and Dr. Brown-Séquard gives a theoretical explanation of their beneficial mode of action. They produce a *similia similibus* effect. We have seen that the disease, according to Dr. Brown-Séquard, results from a contraction of the smaller blood-vessels; but if this contraction be considerable, the muscular fibres of the vessels become exhausted, and then (by a well-established physiological law) they are relaxed, and so there follow dilatation of blood-vessels and admission of due flow of blood to the spine. Now, no irritating cause is more powerful in effecting contraction of blood-vessels by reflex action than cold; and, consequently, this sort of excitation alternated with heat, should be applied to the spine. But, whatever be the kind of excitation employed in this case, it ought to be very powerful, in order to produce that exceeding degree of contractility of muscular fibres which is found, experimentally, to be followed by exhaustion and dilatation.

We fancy that to the theoretical explanation given by Dr. Brown-Séquard (for we must still call it such,) of the cause of reflex paraplegia, our readers will not at present be prepared to give their unqualified adhesion. To us, we must say that a tonic permanent spasm of the blood-vessels, such as is supposed by him to be the permanent state of the blood-vessels of some of the nerves or of the spinal cord in reflex paraplegia, is quite incomprehensible; and we think that on this point, which is assuredly the great point of Dr. Séquard's labors in this direction, he would have done well to have more fully developed the basis of his conclusion. Surely it is not enough to satisfy us on this head, that Dr. Brown-Séquard *has seen* a contraction of the blood-vessels in the spinal cord when a "tightened ligature was placed around the hilus of a kidney." *Permanent* contractions of blood-vessels of this character are surely contrary to what experience teaches us in other cases; and if they really exist, how comes it that the cord does not become organically affected through such a cutting off of its nutritive supply?

Keeping this idea of the cause of the paraplegia in view, Dr. Brown-Séquard recommends that the patient, during the night, and, as far as may be, during the day, should lie in that position which would favor (by gravity) the flow of blood to the spine. As regards general treatment, this should consist in tonics and generous food; the partially paralyzed muscles being as much exercised as possible in the open air.

Strychnia, Dr. Brown-Séquard says, is of the greatest use in this affection. It is the only remedy which really deserves confidence. It increases the amount of blood in the spinal cord, and (acting in a special and direct manner on the tissue of the cord) it increases the reflex faculty; the amount of blood and of the reflex faculty being, as propounded, below par in these cases. It may be given with opium in doses of one-thirtieth to one-fortieth of a grain a day, and when used alone, in daily doses of one-twentieth of a grain. When employed with belladonna its dose must be larger, to compete with the antagonistic action of the belladonna. And hot and ice-cold douches may be applied to the spine alternately to produce that violent degree of spasm of the blood-vessels which is followed by their relaxation.

Besides this, the muscles of the paralysed limbs should be duly exercised, to prevent their becoming wasted and atrophied. It is therefore important to have them galvanized two or three times a week, and the limbs properly shampooed. If necessary, also, the heat of the limbs must be artificially kept up; remembering always that, unless the *primum mobile* of the paralysis—the local irritation—be removed, we can expect no permanent cure.

